# Green synthesis of carbon quantum dots embedded onto titanium dioxide nanowires for enhancing photocurrent

**DOI:** 10.1098/rsos.161051

**Published:** 2017-05-10

**Authors:** Yin-Cheng Yen, Chia-Chi Lin, Ping-Yu Chen, Wen-Yin Ko, Tzu-Rung Tien, Kuan-Jiuh Lin

**Affiliations:** Department of Chemistry, National Chung Hsing University, Taichung 402, Taiwan, Republic of China.

**Keywords:** carbon quantum dot, titanium dioxide, water splitting, peroxide and photo catalyst

## Abstract

The green synthesis of nanowired photocatalyst composed of carbon quantum dots-titanium hybrid-semiconductors, CQDs/TiO_2_, are reported. Where graphite-based CQDs with a size less than 5 nm are directly synthesized in pure water electrolyte by a one-step electrochemistry approach and subsequently electrodeposited onto as-prepared TiO_2_ nanowires through a voltage-driven reduction process. Electron paramagnetic resonance studies show that the CQDs can generate singlet oxygen and/or oxygen radicals to decompose the kinetic H_2_O_2_ intermediate species upon UV light illumination. With the effect of peroxidase-like CQDs, photocurrent density of CQDs/TiO_2_ is remarkably enhanced by a 6.4 factor when compared with that of as-prepared TiO_2_.

## 1. Introduction

Titanium dioxide (TiO_2_) is one of the most attractive photocatalytic materials which has been used extensively for photo electrochemical related applications because of its high photo activity and excellent chemical stability as well as low cost [1,2]. However, current direct TiO_2_ nanomaterials for water splitting face many challenge issues: one is the wild band gap of TiO_2_ which limits the light harvest [3–8]. The other critical problem is the generated peroxide via a two-electron pathway will delay the release of oxygen and poison the activity of TiO_2_ [9–11]. For longstanding application of TiO_2_ on water splitting, it is necessary to enlarge light harvest in the visible region and eliminate peroxides during the water splitting process. Traditionally, chemical doping and dye sensitizing are used to enhance light absorption [12]. Furthermore, metal-catalyst [13–15], chemical agent [16] and physical bubbling methods [17] have been reported for removing the H_2_O_2._ Unfortunately, there is lack of a solution to overcome all problems at once.

Carbon quantum dots (CQDs) are a new kind of carbon nanostructure which have received wide attention owing to their unique properties of good biocompatibility, robust chemical inertness and high resistance to photobleaching [18–23]. Moreover, the optical properties of CQDs can be tuned by size control, chemical doping and functionalization for featured applications [24–27]. Recently, an interesting result was found that CQDs which could serve as peroxidase mimetics demonstrate an excellent catalytic activity on the decomposition of H_2_O_2_ [28]. Liu *et al.* reported that metal-free CQD-carbon nitride nanocomposites serving as photocatalysts were explored to break the peroxide down [9]. These results provide an idea to develop CQD-containing hybrid semiconductors for solving toxic issues during the water splitting process.

Generally, CQDs could be produced by traditional methods such as electrochemical oxidation, laser ablation, hydrothermal/solvothermal treatment, microwave irradiation and arc discharge [24,29]. Recently, a special chemical cleavage of layered N-doped carbon materials, carbon nitride quantum dots (CNQDs), was proposed [30]. Lately, the facile electrochemical fabrication of water-soluble CQDs has been achieved using an alkali-base (NaOH/EtOH) electrolyte [31]. Unfortunately, these processes require chemicals/surface passive agents, complex instrumental set-ups or post-treatments. To the best of our knowledge, one-step synthesis of homogeneously dispersed CQDs in pure water without using chemicals and further post-treatments is still challenged. Herein, we report a straightforward three-electrode electrochemical approach to produce high-quality CQDs in pure water electrolyte (i.e. without using acids and bases). This facial electrochemical fabrication offers a one-step technology to prepare CQD water solution without further post-treatments like filtration, dialysis, centrifugation, column chromatography and gel-electrophoresis. A hybrid photoactive anode that consists of CQDs and titanium dioxide nanowires (CQDs/TiO_2_ NWs) was fabricated for high efficient water splitting via an electrodeposition approach. Results revealed that the CQDs play a role of peroxidase to catalyse the transformation of H_2_O_2_ into oxygen which leads to lower charge transfer resistance and ion diffusion resistance. Therefore, photocurrent density of CQDs/TiO_2_ NWs was remarkably enhanced by a 6.4 factor when compared to that of an as-prepared TiO_2_ NW photo anode.

## 2. Material and methods

### 2.1. Preparation of carbon quantum dot solution

The graphite-coated rod (electron conductivity, 1.25 ohm cm^−1^; hardness, 64 Hs) was inserted into the ultra-pure water (18 M ohm cm^−1^, 30 ml) as the anode, the Ag/AgCl as the reference electrode and the platinum wire as the counter electrode. The synthetic control parameters of cyclic voltammetry (CV) include that scan range of applied potential is +3 V to −3 V and scan rate is 0.5 V s^−1^ with 3500 cycles.

### 2.2. Preparation of CQDs/TiO_2_ NWs on FTO

FTO (F:SnO_2_) (TEC-7, 8 ohm square) was cleaned by ultrasonic agitation in detergent, deionized water and a mixture solution of ethanol, acetone and deionized water with a volume ration of 1 : 1 : 1 for 15 min, respectively. The FTO substrates were immersed in an aqueous of 0.5 M TiCl_4_ (99%, Merck) at 80°C for 30 min and followed by heat treatment at 500°C for 30 min to generate a compact TiO_2_ layer. The TiCl_4_-treated substrates were then suspended in a reagent solution that contained 6 ml 2-butanone (more than 99%, Merck), 6 ml HCl (12 M, Merck) and 0.6 ml tetrabutyl titanate (more than 97%, Alderich) in a Telflon vessel. The telflon vessel was sealed in an autoclave and heated at 200°C for 1.5 h. After further annealing at 500°C for 30 min, the crystallinity of TiO_2_ NWs was grown on FTO substrates. The electrodeposition of CQDs onto TiO_2_ NWs was performed with a standard three-electrode system (electronic supplementary material, figure S1), consisting of a TiO_2_ NW electrode as the working electrode, Ag/AgCl as the reference electrode and platinum sheet as the counter electrode, at –3.0 V for 1 h.

### 2.3. Characterizations

The morphology and lattice spacing images of CQDs and CQDs/TiO_2_ NWs were recorded by transmission electron microscopy (TEM, JEM-2010, JEOL) operated at 200 kV. The absorbance spectra were obtained with a UV/vis/NIR spectrometer (Lambda 900, Perkin Elmer). The photoluminescence (PL) spectra were performed using a spectrofluorometer (FP-6200, JASCO). The crystal structure was characterized by Raman spectroscopy (Nanofinder 30, Tokyo Instruments, INC) with a 488 nm laser. The Fourier transform infrared (FTIR) spectroscopy spectra were recorded using a FTIR spectrometer (BRUKER/Vertex 70 V). X-ray photoelectron spectroscopy (XPS) data were obtained with a ULVAC-PHI, PHI 5000 VersaProbe/Scanning ESCA Microprobe. Fitting of the XPS data was accomplished using XPSPEAK41 software. The measurement of photocurrent was carried out using a three-electrode system. TiO_2_ NW electrode with or without CQDs was the working electrode; an Ag/AgCl electrode in 3 M KCl was the reference electrode; the Pt sheet was used as the counter electrode. All electrodes were examined in 0.5 M Na_2_SO_4_ solution with a PARSTAT 2263 Advanced Electrochemical System under illumination using a Newport solar simulator with AM 1.5 G (100 mW cm^−2^). Before the experiment, the electrolyte was purged by argon to remove the dissolved oxygen. The SigmaScan Pro 5 software was used to calculate the particle size of CQDs and the measured area of working electrode for calculating photocurrent density. The electrochemical impedance spectroscopy (EIS) was measured by applying the open-circuit voltage under 1 sun illumination and recorded over the frequency range of 200 kHz–100 mHz with ac amplitude of 10 mV by using a PARSTAT 2263 Advanced Electrochemical System. EIS results were analysed and fit using the software program ZView.

## 3. Results and discussion

**Figure 1.**
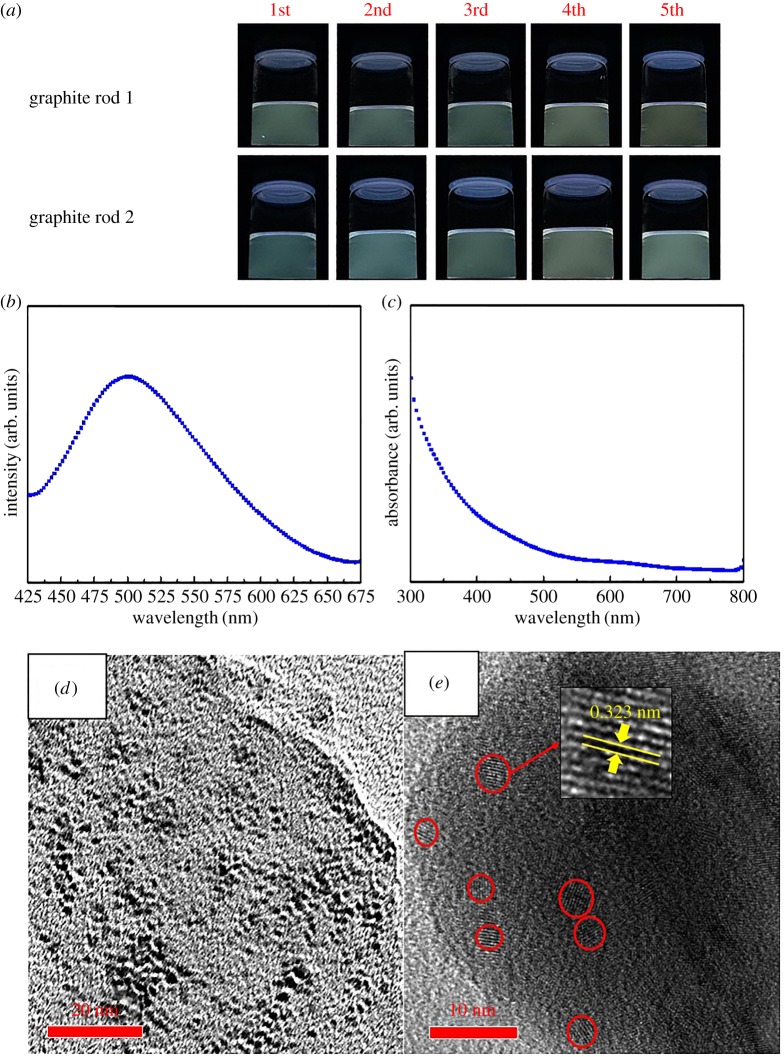
(*a*) Photos of as-prepared CQD water solution under UV light (365 nm), in which the sample was obtained after 3500 CV scan cycles on two types of graphite-coated rods. (*b*) UV-Vis spectrum and (*c*) PL spectrum of CQD solution. (*d,e*) HRTEM images of CQDs.

The photograph of CQD solution is show in figure 1*a*. After 3500 scan cycles, the colour of the CQD's solution is probably clear; however, a pale-green emission appeared gradually under UV light (365 nm) irradiation in the reactor. We have shown that the present one-step methodology is scalable, repeatable and of straightforward characterizations. The aqueous CQDs exhibited a typical UV-Vis absorption from 300 to 600 nm and a broad emission peaking at 500 mm, while excitation at 365 nm (figure 1*b*,*c*). Remarkably, the corresponding photoelectric properties of CQD solution remain unchanged after storing for 10 months in air at room temperature (electronic supplementary material, figure S2). From HRTEM images, as show in figure 1*d*,*e*, the size distribution of CQDs is from 0.5–4 nm and it presents an average lattice spacing of CQDs as 0.323 nm, corresponding to (002) planes of graphite [32,33].

**Figure 2.**
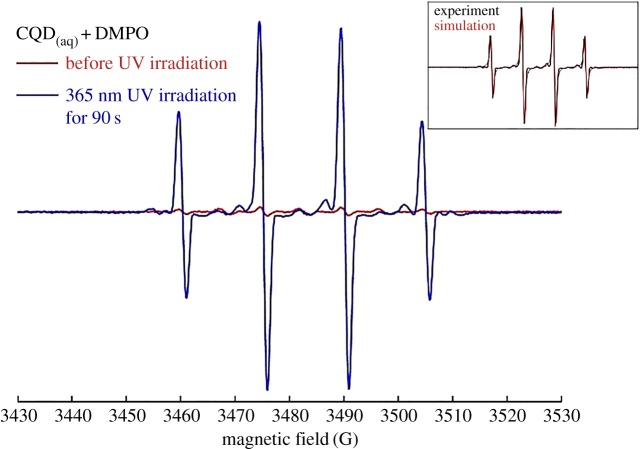
EPR signals of hydroxyl radical obtained under irradiation of 365 nm on sp^2^-CQD solution in the presence of DMPO for 90 s. Inset: simulation of this signal after irradiation by EasySpin toolbox in Matlab software with magnetic parameters: *g* = 2.0051; *A*_N_ = 14.85 G; and *A*_H_ = 15.12 G. Instrumental settings for detection: frequency, 9.774 GHz; power, 5.044 mW; receiver gain, 8.93 × 10^−3^; modulation amplitude, 2 G; time constant, 40.96 ms; resolution, 2048 points.

It was reported that small graphite-like nanoparticles revealed excellent up-conversion luminescence properties and even generated singlet oxygen (^1^O_2_) and/or oxygen radicals from water [34–36]. Accordingly, electron paramagnetic resonance (EPR) was employed to probe ^1^O_2_ in CQD solution under UV irradiation by means of spin trap reagent, 5,5-dimethyl-1-pyrroline N-oxide (DMPO), which has been proposed to detect ^1^O_2_ by readily forming [DMPO−^1^O_2_] intermediately succeeded by quick decomposition into DMPO–OH radical as well as the equivalent hydroxyl radical [37]. The EPR spectra are highlighted in figure 2. Weak signals are observed before UV irradiation because of slight oxidation of DMPO arose by ambient visible light during sample preparation. Upon irradiation of 365 nm for 90 s, a highly intense signal with typical 1 : 2 : 2 : 1 DMPO/•OH peaks is consequently obtained, indicating that the extremely efficient production of ^1^O_2_ and/or oxygen radicals is facilely induced by photo-activated CQDs in water solution.

**Figure 3.**
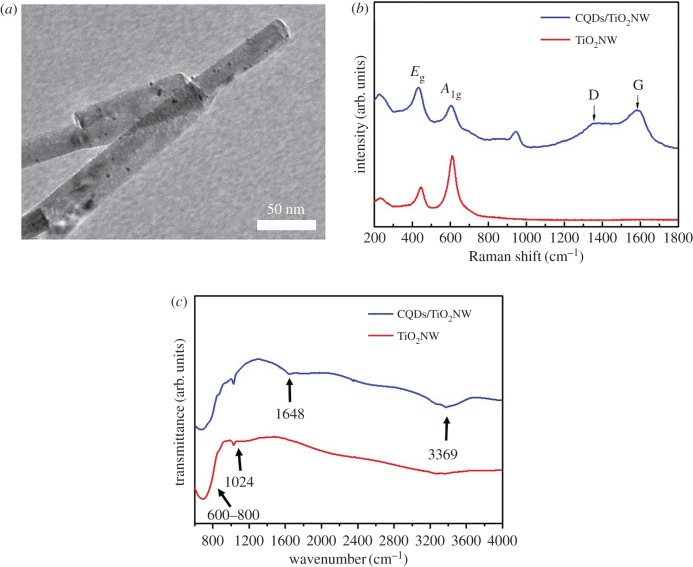
(*a*) HRTEM of CQD/TiO_2_ NWs; (*b*) Raman spectra; and (*c*) FTIR spectra of TiO_2_ NWs and CQDs/TiO_2_ NWs.

**Figure 4.**
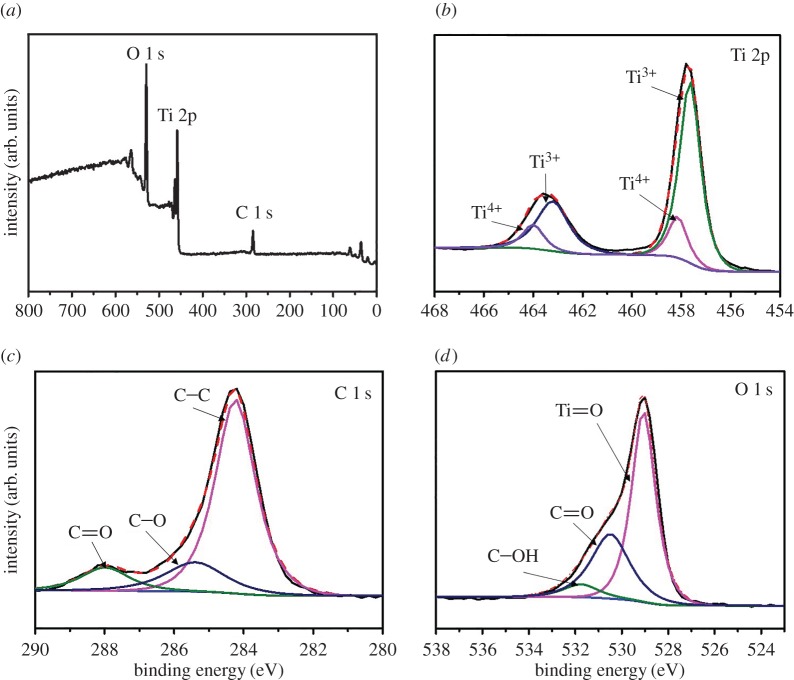
XPS spectra of CQDs/TiO_2_ NWs. (*a*) Full survey. (*b*) Ti 2p spectra. (*c*) C 1 s spectra. (*d*) O 1 s spectra.

To further explore the photo-converse-electron efficiency of the CQD-embedded titanium dioxide system, we offer the voltage-driven reduction process for the growth of CQDs/TiO_2_ NWs. An HRTEM image of the growth of CQDs onto TiO_2_ NWs while electrodepositing at −3 V for 1 h is shown in figure 3*a*. It was observed that TiO_2_ NWs have a diameter of 30 nm and the dark dots in the TEM image represent the CQDs. Moreover, the distance between the adjacent lattice fringes is 0.325 nm and 0.223 nm, which can be assigned to the interplane distance of the rutile TiO_2_ (110) plane and (101) planes of graphite (JCPDS card: no. 65-6212) (electronic supplementary material, figure S3). Further characterization studies of Raman spectra provided convincing evidence for the graphite-based structure of CQDs on TiO_2_ NWs (figure 3*b*). Dominant peaks at 233, 446 and 610 cm^−1^ are assigned to the Raman active modes of rutile-phased TiO_2_. The G-band at 1587 cm^−1^ attributes to the *E*_g_ mode of the graphite and is associated with sp^2^-bonded configuration, whereas a broad D-band at 1355 cm^−1^ corresponds to the vibration modes of sp^3^-carbon atoms of disordered graphite [26,38]. FTIR spectra of the CQDs/TiO_2_ NWs are shown in figure 3*c*. The peak for Ti-O-Ti and Ti-O was present in the range of 600–800 and 1024 cm^−1^, respectively [39,40]. The characteristic transmittance peaks of the CQDs/TiO_2_ NWs at 1648 cm^−1^ associated with the C=O stretching vibration, indicate the existence of CQDs in the composites [33]. Moreover, the broad peak at 3369 cm^−1^ was attributed to vibrations of surface adsorbed water [41]. XPS was carried out to investigate the components and surface properties of CQDs/TiO_2_ NW composite which is shown in figure 4. The full survey spectrum of figure 4*a* indicates the presence of titanium (Ti 2p), carbon (C 1 s), and oxygen (O 1 s) in the CQDs/TiO_2_ NW composites. The Ti 2p spectra were deconvoluted and resolved into four spin orbit components at 2p3/2 binding energies 457.6, 458.14 eV and their corresponding 2p1/2 components (463.21, 464.0 eV) which are assigned as Ti^3+^ (TiOOH/coordinatively unsaturated) and Ti^4+^ (TiO_2_), respectively [42,43]. The large ratio of Ti^3+^ is suggested owing to the increased electron content of the lattice surface of TiO_2_ within the voltage-driven reduction process [44]. The peaks at 284.24, 285.44 and 287.99 eV for C 1 s spectrum are ascribed to the C–C bond with sp^2^ orbital, C–O and C=O bonds, respectively. The main peak of O 1 s spectrum at 529.06, 530.49 and 531.49 eV is attributed to Ti=O, C=O and C–OH, respectively [45,46].

**Figure 5.**
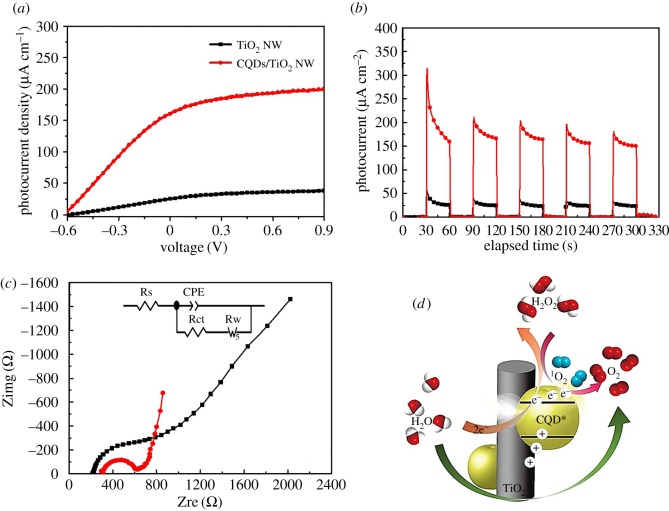
(*a*) LSV curve (*b*) *I*–*t* curve and (*c*) Nyquist plot of TiO_2_ NWs and CQDs/TiO_2_ NWs under AM 1.5 G sunlight illumination. (*d*) Proposed scheme of decomposition of H_2_O_2_ by peroxidase-like CQDs on TiO_2_ NWs. (EPR study of the decomposition of H_2_O_2_ by CQDs was illustrated in the electronic supplementary material, figure S4.)

Measurements of linear sweep voltammogram (LSV) and photocurrent density versus elapsed time (*I–t*) of CQDs/TiO_2_ NWs and as-prepared TiO_2_ NW devices were carried out under AM 1.5 G sunlight illumination, as shown in figure 5*a*,*b*. It is important to note that a photocurrent density of 160 μA cm^−2^ at 0 V versus Ag/AgCl for CQDs/TiO_2_ NWs was remarkably obtained and a net enhancement ratio of 6.4 was achieved when compared with the as-prepared TiO_2_ electrode (25 µA cm^−2^). From figure 5*b*, both of two photoanode devices represented good reproducibility and stability as the illumination was continually turned on and off. Furthermore, the sharp spike in the photocurrent during the on/off illumination cycles demonstrates the predominant transport of photo-generated electron in the CQDs/TiO_2_ NWs. The increased photocurrent of CQDs/TiO_2_ NWs may be attributed to the effect of synergetic photocatalytic behaviour and peroxidase-like property of CQDs which facilitate charge separation/transportation during the water splitting process. In order to verify our suggestions, the study of EIS of interfacial electron transfer of CQDs/TiO_2_ NWs is highlighted in figure 5*c*. EIS spectra show that both photoanodes revealed an obvious semicircle of Nyquist plot at the high frequencies and a Warburg type line at low frequencies under illumination [47]. The equivalent circuit modes applied to fit the experimental EIS data of the as-prepared TiO_2_ NWs and the CQDs/TiO_2_ NW composite electrode is shown in the inset of figure 5*c*. The fitting results of all parameters of the equivalent circuit are listed in table 1. It should be noted that a larger charge transfer resistance (Rct) for TiO_2_ electrode (520.6 Ω) was observed when compared with that of the CQDs/TiO_2_ NW electrode (337.9 Ω). In addition, the Warburg resistance for the CQDs/TiO_2_ NW electrode (1273 Ω) is apparently smaller than that of the TiO_2_ electrode (4921 Ω). From EIS analysis, it demonstrated that CQDs/TiO_2_ NWs exhibit predominant electron transport property and ionic conduction at the interfacial heterojunction of CQDs/TiO_2_ NWs and it could be attributed to the synergetic photocatalytic behaviour and peroxidase property of CQDs.

**Table 1.** EIS fitting parameters of as-prepared TiO_2_ NWs and CQDs/TiO_2_ NWs.

description	parameters	TiO_2_ NWs	CQDs/TiO_2_ NW	unit
Ohmic series resistance	Rs	216.9	304.9	Ω
charge transfer resistance at electrode/electrolyte interface	Rct	520.6	337.9	Ω
CPE for capacitance of electrode	CPE	1.89×10^−5^	1.90×10^−6^	F · s*^*n*^*^−1^
Warburg impedance	Rw	4921	1273	Ω

The proposed scheme of decomposition of H_2_O_2_ by peroxidase-like CQDs is shown in figure 5*d*. Upon solar illumination on CQDs/TiO_2_ NWs, photo-generated carriers were separated into photo-generated electrons (e^−^) and photo-generated holes (h^+^). The e^−^ and h^+^ react with adsorbed oxidants/reducers (O_2_/OH^−^) to produce photo-reactive oxygen radicals (^1^O_2_, •OH) which play the key role in the decomposition of the H_2_O_2_ intermediates, which is in good agreement with the EPR study (electronic supplementary material, figure S4) [48,49].

## 4. Conclusion

In summary, for the first time to our knowledge, the CQDs with a size less than 5 nm in water solution have been synthesized by the one-step electrochemical method without using chemicals and further post-treatments. For enhancing the water splitting photocurrent, CQDs/TiO_2_ NWs are used to construct a synergetic semiconductor as a hybrid photoanode. A remarkable enhancement by 6.4 times on water splitting current was observed which is attributed to CQDs which served as an efficient catalyst for decomposing H_2_O_2_, resulting in a reduced interfacial charge transferred resistance and fast ion diffusions. The green synthesis CQDs and CQDs/TiO_2_ NWs may open a new way to design a broad range of photoelectric materials for application in bioscience and new energy-saving technology.

## Supplementary Material

Figure S1; Figure S2; Figure S3; Figure S4

## Data accessibility

The datasets supporting this article have been uploaded as part of the electronic supplementary material.
