# Supplementary material for: Green synthesis of carbon quantum dots embedded onto titanium dioxide nanowires for enhancing photocurrent
Source: R Soc Open Sci. 2017 May 10;4(5):161051. doi: 10.1098/rsos.161051 (PMC5451797; doi:10.1098/rsos.161051)
Supplement: Figure S1; Figure S2; Figure S3; Figure S4 [file rsos161051supp1.docx]

**Electronic Supplementary Information**

**Green Synthesis of Carbon Quantum Dots Embedded onto Titanium Dioxide Nanowires for Enhancing Photocurrent**

Yin-Cheng Yen, Chia-Chi Lin , Ping-Yu Chen, Wen-Yin Ko, Tzu-Rung Tien and Kuan-Jiuh Lin*

Department of Chemistry, National Chung Hsing University, Taichung 40227, Taiwan

[Corresponding author*]

Mail address: Department of Chemistry, National Chung Hsing University, 250 Kuo-Kuang Rd., Taichung, 402 Taiwan, R.O.C.

E-mail: kjlin@dragon.nchu.edu.tw

Phone: +886-4-22840411#802

Fax: +886-4-22854488


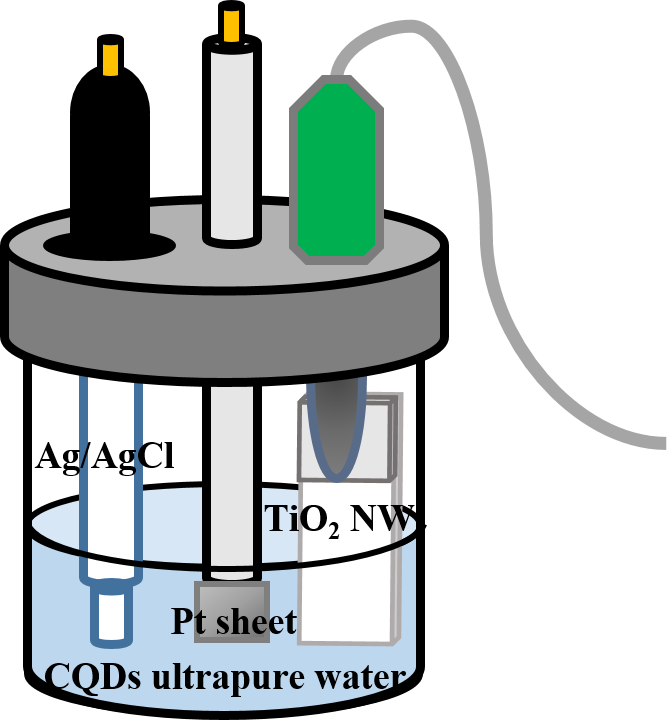


**Figure S1.** Three electrode system for fabrication CQDs/TiO_2_ NW by electrodeposition.


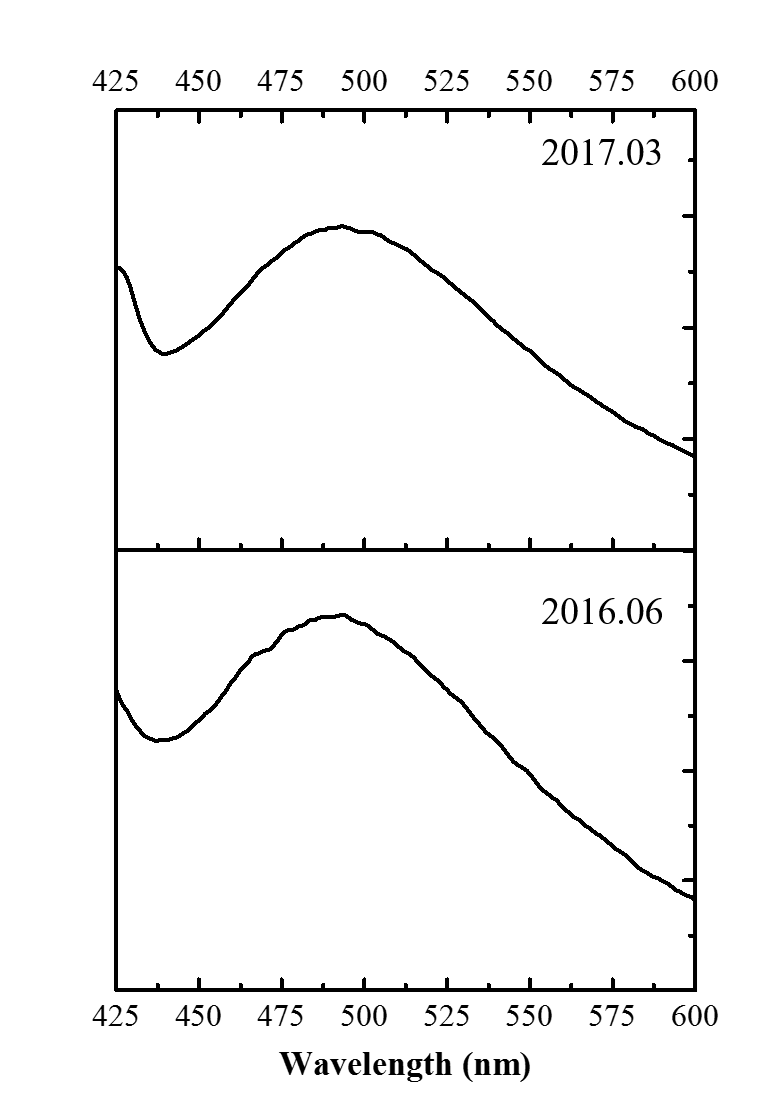


**Figure S2.** PL spectra of CQD solution on 2016.06 and 2017.03.


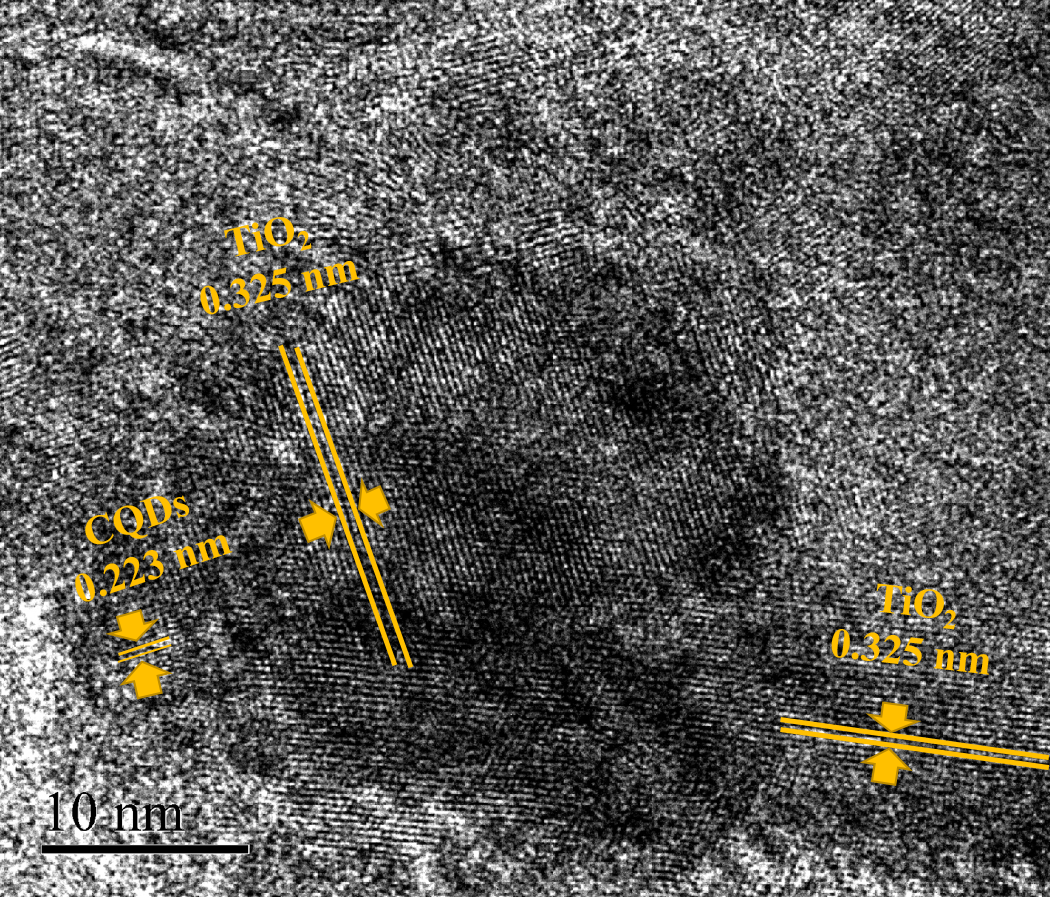


**Figure S3.** HRTEM images of CQDs/TiO_2_ NW.


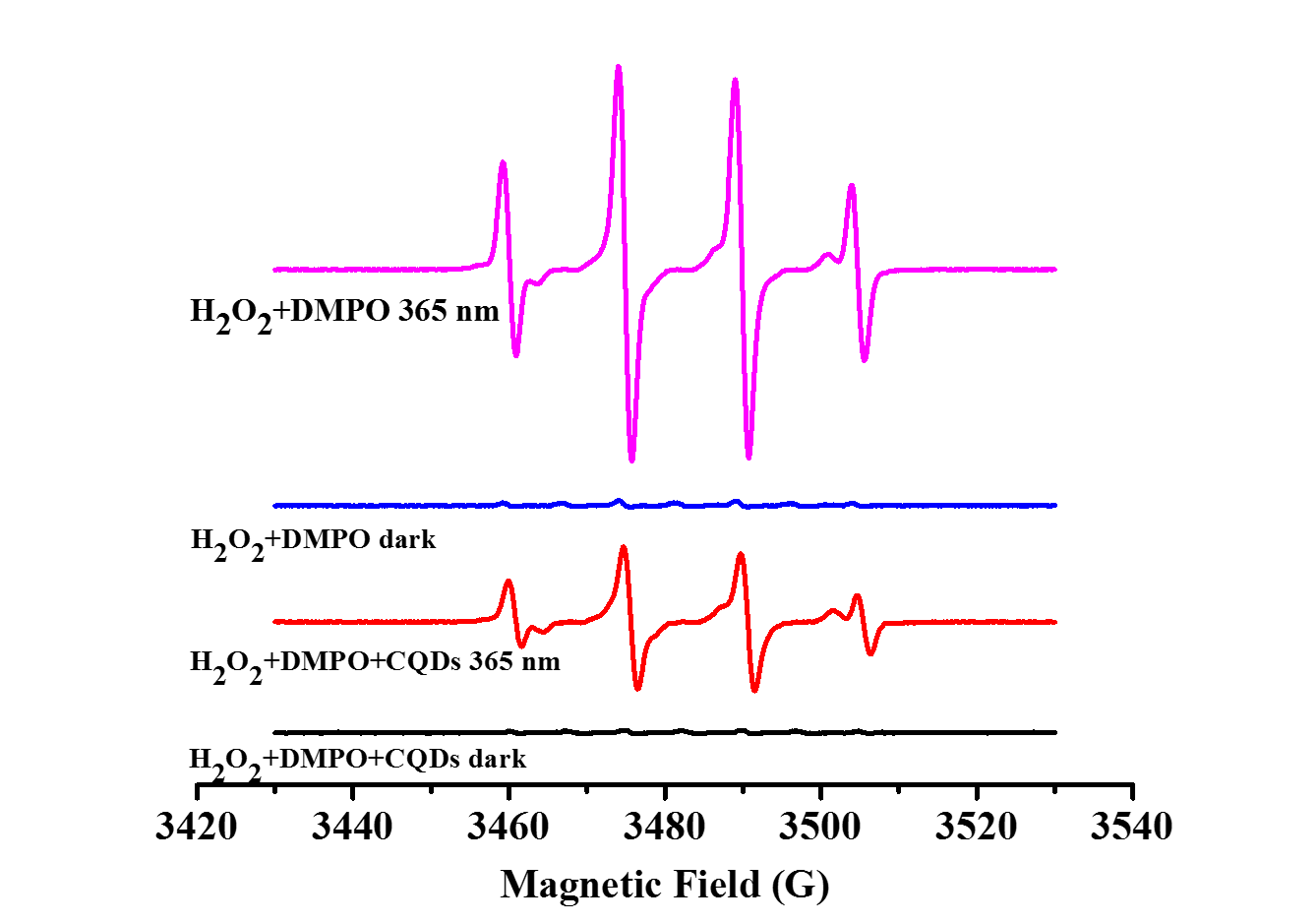


**Figure S4.** EPR signals of hydroxyl radical obtained under irradiation of 365 nm on solution in the presence of DMPO for 90 seconds.

EPR was employed to probe hydroxyl radical (･OH) in H_2_O_2_ and CQDs/H_2_O_2_ solution under UV irradiation by means of spin trap reagent, 5,5-dimethyl-1-pyrroline N-oxide (DMPO). Under illumination, H_2_O_2_ will photolysis into ･OH. Therefore, a highly intense signal with typical 1:2:2:1 DMPO/･OH peaks are determined. With adding CQDs into H_2_O_2_ solution, the signal intensity of DMPO/･OH was reduced, suggesting that CQDs reduce the ･OH radical generation by catalyzing the H_2_O_2_ decomposition into O_2_ and H_2_O.
